# The lack of autophagy triggers precocious activation of Notch signaling during *Drosophila* oogenesis

**DOI:** 10.1186/1471-213X-12-35

**Published:** 2012-12-05

**Authors:** Julia MI Barth, Ernst Hafen, Katja Köhler

**Affiliations:** 1 Institute of Molecular Systems Biology, ETH Zurich, Zurich, Switzerland

**Keywords:** Autophagy, *Drosophila*, Follicle cells, Oogenesis, Notch

## Abstract

**Background:**

The proper balance of autophagy, a lysosome-mediated degradation process, is indispensable for oogenesis in *Drosophila*. We recently demonstrated that egg development depends on autophagy in the somatic follicle cells (FC), but not in the germline cells (GCs). However, the lack of autophagy only affects oogenesis when FCs are autophagy-deficient but GCs are wild type, indicating that a dysfunctional signaling between soma and germline may be responsible for the oogenesis defects. Thus, autophagy could play an essential role in modulating signal transduction pathways during egg development.

**Results:**

Here, we provide further evidence for the necessity of autophagy during oogenesis and demonstrate that autophagy is especially required in subsets of FCs. Generation of autophagy-deficient FCs leads to a wide range of phenotypes that are similar to mutants with defects in the classical cell-cell signaling pathways in the ovary. Interestingly, we observe that loss of autophagy leads to a precocious activation of the Notch pathway in the FCs as monitored by the expression of Cut and Hindsight, two downstream effectors of Notch signaling.

**Conclusion:**

Our findings point to an unexpected function for autophagy in the modulation of the Notch signaling pathway during *Drosophila* oogenesis and suggest a function for autophagy in proper receptor activation. Egg development is affected by an imbalance of autophagy between signal sending (germline) and signal receiving cell (FC), thus the lack of autophagy in the germline is likely to decrease the amount of active ligand and accordingly compensates for increased signaling in autophagy-defective follicle cells.

## Background

Autophagy is a tightly regulated, lysosomal degradation process occurring in all eukaryotic cells from yeast to mammals. Under normal induction, as for example during cellular stress, unnecessary cytosolic components are recycled to promote cell survival. However, autophagy can also lead to programmed cell death and is needed throughout normal development. Furthermore, it plays a role in immunity, lifespan extension and many human diseases, such as neurodegeneration and cancer [1].

In *Drosophila*, autophagy plays a crucial role during metamorphosis to remodel larval tissues such as the fatbody and salivary glands, and starvation triggers autophagy in nutrient responding organs, e.g. the fatbody and the ovaries [2-5]. During oogenesis, nutrient depletion induces autophagy at several “check points”. First, in region 2b in the germarium, both autophagy and apoptosis can be detected during normal development, but are increased under starvation conditions [2,6-8]. Similar observations have been made for the second check point during mid-oogenesis, where degenerating egg chambers display autophagic markers and eggs composed of an autophagy-deficient germline are impaired to activate autophagy, but also DNA fragmentation, which is denotive for apoptosis [7-10].

In addition to starvation-induced autophagy, developmental autophagy also occurs in germ cells (GCs) and follicle cells (FCs) during oogenesis. Late stage FCs undergo cell death after chorion deposition, showing the appearance of autophagic structures and condensed chromatin, but no DNA fragmentation, which suggests a mechanism independent of caspases [2,11]. Further, it was shown that developmental cell death in region 2b of the germarium and during mid-oogenesis, as well as the nurse cell death occurring in late oogenesis depend on autophagy [8,10]. In line with these findings, Nezis et al. demonstrated that autophagosomal markers accumulate in dying stage 13 nurse cells, and that egg chambers containing germline clones (GLC) mutant for the autophagy-related genes *(ATG) ATG1*, *ATG13* or *Vps34* showed no DNA fragmentation, but persisting nurse cells nuclei (PNCN), suggesting that autophagy is essential in the germline [12]. However, we have recently demonstrated that autophagy-deficient GCs give rise to normal eggs without the appearance of PNCNs. In contrast, *ATG* gene deficiency in the FCs led to defective eggs, indicating that autophagy is specifically required in the FCs to support proper egg development [2].

Interestingly, autophagy deficiency only affects oogenesis in a cellular context where FCs are mutant for *ATG* genes but GCs are wildtype (WT), indicating that a dysfunctional signaling between soma and germline may be responsible for the oogenesis defects [2]. During egg development, several classical signaling pathways are shared between the GCs and FCs and are essential for cell differentiation and axis specification [13]. For example, Gurken protein translated by the oocyte activates epidermal growth factor receptor (EGFR) signaling in the adjacent terminal FCs, defining them as posterior FCs [14]. In turn, a yet unknown signal from the newly defined posterior FCs to the oocyte (back signaling) triggers the movement of the oocyte nucleus from the posterior side to an asymmetrical anterior position, which subsequently will be defined as the dorsal side of the egg chamber by a second round of Gurken/EGFR signaling from the oocyte to the overlaying FCs [14,15]. On the other hand, signaling of the ligand Delta (expressed by the germline) to the Notch receptor (expressed by FCs) leads to differentiation of polar cells in early stages, a switch from the mitotic to an endoreplication program during mid-oogenesis and the correct differentiation of dorsal appendage (DA) roof and floor cells in late oogenesis [16-18]. For both pathways, EGFR and Delta-Notch, endocytosis and endosomal trafficking is required within ligand and/or receptor presenting cells for activation, regulation and degradation of the signal [19,20].

In this study, we extend our analyses on the role of autophagy during oogenesis and show that eggs composed of FCs mutant for *ATG* genes exhibit phenotypes similar to mutants with defects in the classical cell-cell signaling pathways in the ovary. Furthermore, we could designate specific FC subpopulations that are involved in the autophagy-dependent control of egg development by using spatially restricted interfering RNA (RNAi) mediated knock down. Finally, we demonstrate that autophagy modulates the expression of the Notch downstream targets Cut and Hindsight, implying a precocious activation of the pathway. These results reveal a novel function of autophagy and open exciting opportunities to examine the influence of autophagy on receptor/ligand regulation. According to our model in which autophagy affects oogenesis only when FCs are *ATG* mutant and the germline is WT, we propose that autophagy deficiency in the germline may reduce the abundance of active ligand to compensate for increased receptor signaling in the autophagy-defective signal receiving cells.

## Results

### Ovaries lacking *ATG* gene function in the FCs exhibit multiple egg chamber defects

**Figure 1:**
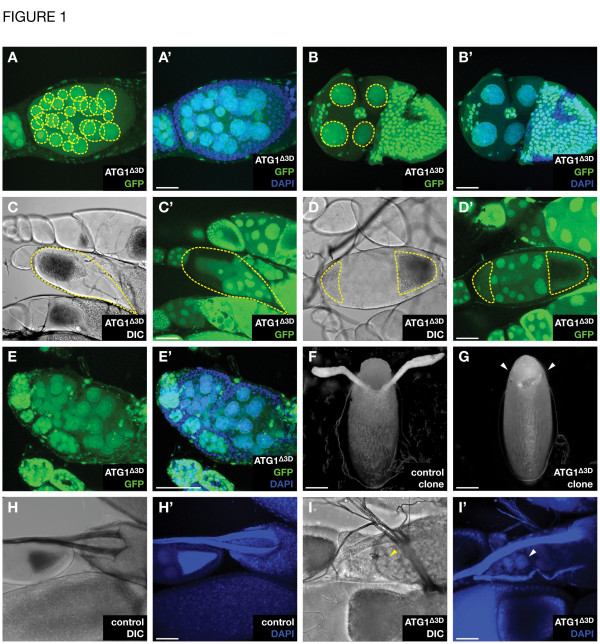
**Lack of *ATG1 *in FCs affects proper egg development.** Heat shock flipase (HS-FLP) mediated generation of FC clones mutant for *ATG1* (marked by the lack of GFP) caused a wide array of oogenesis defects. Compound eggs with more than 16 cysts (outlined in yellow) (**A**, **A’**) and ovarioles lacking stalk cells (**E**, **E’**) were often observed. Some eggs consisted of less than the 16 germline cyst normally present in WT eggs (outlined in yellow) (**B**, **B’**). Inverted eggs presenting the oocyte in the wrong position (outlined in yellow) (**C**, **C’**) or eggs with two oocytes (outlined in yellow) (**D**, **D’**) were less frequently observed. Mature eggs regularly lack DAs (arrowheads) (**F**, **G**). Stage 14 egg chambers often contain persisting nurse cell nuclei (PNCN) (arrowheads) (**I**, **I’**) that are normally degenerated in control eggs with WT clones (**H**, **H’**). Anterior is to the left, posterior to the right, except F, G: anterior to the top, posterior to the bottom, dorsal to the front. Scale bar: 50 μm Genotypes: A-E, G and I: *hs flp/+; ATG1*^*∆ 3D*^*FRT80B/FRT80B-UbiGFP*. F and H: *hs flp/+; FRT80Biso/FRT80B-UbiGFP* .

During *Drosophila* oogenesis, slight disturbances interfere with the precise control of egg development, leading to misshaped egg chambers and malformed mature eggs [21]. Accordingly, generating *ATG1* mutant FC clones with the FLP-FRT method resulted in multiple egg chamber defects. Most prominently, egg chambers showed an abnormal number of germline cysts. In many of the egg chambers containing *ATG1* mutant FC clones, we observed more than the 16 cell cysts normally present in WT egg chambers (31.3 +/-6.0% in *ATG1* mutant versus 3.4 +/-2.9% in WT eggs, total number of ovaries counted were 67 and 55, respectively, Figure 1A, A’), whereas an unusual reduction in cyst number was detected less frequently (Figure 1B, B’). Eggs presenting more germline cysts are composed of two fused egg chambers (compound egg chambers) where the mutant FCs have not migrated between the germline cysts. Similarly, the generation of *ATG13* mutant FC clones also resulted in compound egg chambers, albeit at a lower frequency (data not shown). In some cases, single egg chambers within one ovariole showed a wrong orientation (Figure 1C, C’) or featured two oocytes (Figure 1D, D’), and various ovarioles containing *ATG1* mutant FCs were lacking stalk cells that normally interconnect the egg chambers (Figure 1E, E’). Furthermore, we have previously shown that mature eggs with *ATG1* and *ATG13* mutant FC clones often display missing, shortened or malformed DAs (Figure 1F, G, arrowheads) [2]. Many stage 14 egg chambers also displayed persisting nurse cell nuclei (PNCN) (Figure 1 H-I’, arrowheads), a phenotype previously described for *ATG* mutant germline clones (GLCs) achieved with the *Ovo*^*D*^ system (62% and 60% of the eggs containing GLCs mutant for *ATG1* and *ATG13*, respectively) [12]. However, in stage 14 eggs with an *ATG1* or *ATG13* mutant germline generated by pole cell transplantations, we did not detect PNCNs (Table 1) [2]. These varying findings may be explained by the different methods used. The flipase recognition target (FLP-FRT) mediated *Ovo*^*D*^ technique produces mutant GCs, but also clones in the somatic tissue and thus in the FCs (Additional file 1: Figure S1), whereas in the pole cell transplantation experiments, GCs are mutant, but FCs are entirely WT [2]. Therefore, we suggest that *ATG* mutant FC clones induced by the *Ovo*^*D*^ system are responsible for the presence of PNCNs in those eggs. In fact, both heat-shock (HS) FLP (which also generate occasional germline clones, see Barth et al., 2011) and e22c-FLP (which exclusively lead to FC clones, see Duffy et al., 1998) induced *ATG1* or *ATG13* mutant FC clones produce PNCNs (Table 1). Therefore, we suggest that the defect in nurse cell nuclei clearance observed in the *Ovo*^*D*^ experiments may be- at least in part- due to the lack of autophagy in the FCs rather than in the GCs. During late oogenesis, nurse cells (NCs) transport their cytoplasm to the oocyte and undergo programmed cell death required for normal egg maturation [10,12]. Since surrounding FCs act as non-professional phagocytes and take up remnants of the dying NCs, it is likely that autophagy deficiency leads to an incomplete clearance of inclusion bodies and thus PNCN [22,23]. However, PNCN occur both in eggs with an *ATG* mutant or WT germline, as long as the FCs are autophagy deficient (data not shown), indicating that this phenoytpe most likely does not result from an imbalance of autophagy between germline and soma.

**Table 1:** Quantification of persisting nurse cell nuclei comparing different methods used

	**Persisting NC nuclei (PNCN)**		
**Genotype**	**GC mutant (PCT)**	**FC mutant (HS-FLP induced)**	**FC mutant (e22c-FLP induced)**
	%	%	%
*ATG1*^*∆ 3D*^	3.7 +/- 3.6	41.2 +/- 2.9	57.2 +/- 6.8
control	1.2 +/- 2.2	11.0 +/- 2.2	5.9 +/- 1.0
*ATG13*^*∆ 74*^	0 +/- 0	36.5 +/- 2.9	24.4 +/- 4.2
control	0 +/- 0	7.2 +/- 2.8	1.5 +/- 2.2

Total number of egg chambers counted: PCT, *ATG1*^*∆ 3D*^: 144; control: 132; *ATG13*^*∆ 74*^: 89; control: 79. HS-FLP, *ATG1*^*∆ 3D*^: 171; control: 269; *ATG13*^*∆ 74*^: 153; control: 135. e22c-FLP, *ATG1*^*∆ 3D*^: 56; control: 84; *ATG13*^*∆ 74*^: 93; control: 86. Abbreviations: PCT, pole cell transplantation; HS-FLP, heat-shock flipase.

Taken together, this selection of phenotypes in eggs containing *ATG* mutant clones solely in the FCs in combination with our published data on the importance of the autophagic balance between GCs and FCs support a role for FC-dependent autophagy in regulating oogenesis in *Drosophila*[2].

### Specialized FC subpopulations are responsible for the autophagy-dependent DA defects

**Figure 2:**
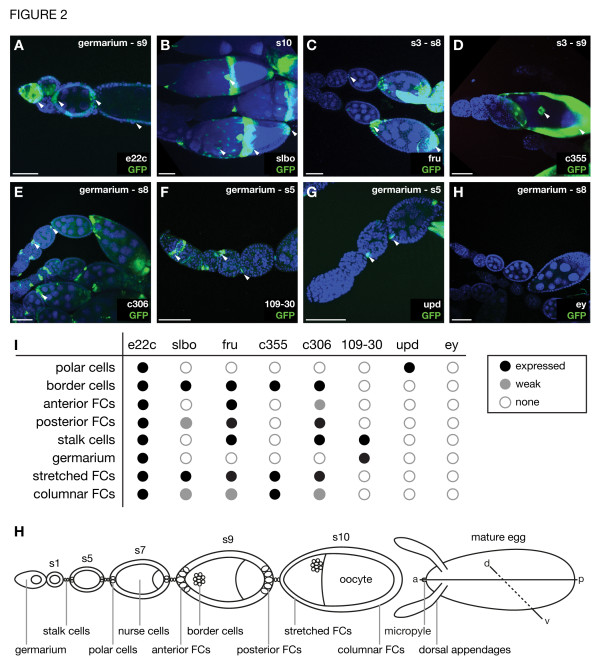
**Expression pattern of different GAL4 driver lines.** The GAL4 driver lines were tested for the expression of UAS-GFP. **A**) The *e22c-GAL4* line drives GFP expression in the follicular stem cells and thus in all FCs, albeit patchy. **B**) *Slbo-GAL4* expresses strongly in border cells, in stretched FCs and in columnar FCs at the dorsal anterior side (centripetal FCs) and the posterior end and also in posterior FCs at early stage 9. **C**) *Fru-GAL4* drives expression in stalk cells, in anterior and posterior FCs from stage 6/7 on, in border cells, stretched FCs and in the very posterior columnar FCs. **D**) *c355-GAL4* expresses from stage 7 onwards in all cells including border, stretched, and columnar cells, but not in polar cells. **E**) *c306-GAL4* expresses in stalk cells, weakly in anterior- and stronger in posterior FCs, border cells, stretched cells and columnar FCs, similar to *slbo-GAL4*. **F**) *109-30*-GAL4 drives expression in stalk precursor- and stalk cells. **G**) *Upd-GAL4* expresses exclusively in the polar cells. **H**) *Ey-GAL4* served as a control and does not drive expression in the ovaries. **I**) Summary of the expression patterns of the GAL4 lines used. **J**) Schematic drawing showing the position of all cell types specified above during different stages of oogenesis. Anterior/posterior FCs corresponds to the stages 6/7 to early 9, stretched and columnar to stages 9 till stage 10/11. Anterior is to the left, posterior to the right. Abbreviations: a, anterior; p, posterior; d, dorsal; v, ventral; s, stage. Scale bar: 50 μm. Genotypes: A: *e22c-GAL4/UAS>GFP*, B: *slbo-GAL4/UAS>GFP*, C: *fru(168)-GAL4/UAS>GFP*, D: *c355-GAL4/+; UAS>GFP/+*, E: *c306-GAL4/+; UAS>GFP/+*, F: *109-30-GAL4/UAS>GFP*, G: *upd-GAL4/+; UAS>GFP/+*, H: *ey-GAL4/UAS>GFP* .

The phenotypes observed in eggs composed of *ATG1* mutant FCs point to an essential function of autophagy in the modulation of a signal transduction pathway during ovary development. As the performance and impact of many signaling pathways is restricted to specific FC subpopulations, we aimed to localize the FC type that is responsible for the defects observed in eggs containing *ATG* mutant FCs. Thus, spatially defined GAL4 driver lines to express RNAi against different *ATG* genes were used to knock down autophagy [24]. To verify the specific activity of the GAL4 lines in certain FC subpopulations, we first documented the expression pattern using a *UAS>GFP* construct (Figure 2). The broadest pattern, showing expression in nearly all FCs from the somatic follicular stem cells until late oogenesis, was observed using the *e22c-GAL4* driver line (Figure 2A and I) [25]. Slow border cell GAL4 (*slbo-GAL4*) is expressed in the border cells, a group of cells that travels together with the anterior polar cells to the oocyte and later forms the micropyle (Figure 2J) [26]. It is also expressed in stretched and columnar FCs at the dorsal anterior side (centripetal FCs) and the posterior end of the egg chamber and can already be detected in posterior FCs at early stage 9. (Figure 2B, I, J) [27]. Fruitless GAL4 (*fru-GAL4*) is expressed in the interconnecting stalk cells, in anterior and posterior FCs starting from stage 6, in border cells, stretched FCs and in very posterior columnar FCs (Figure 2C, I, J). The *c355-GAL4* driver is expressed from stage 7 onwards in all cells including border, stretched and columnar cells, but not in polar cells (Figure 2D, I, J) [28]. *c306-GAL4* drives expression in stalk cells, weak in anterior FCs, stronger in posterior FCs, border cells, stretched cells and columnar FCs similar to slbo (Figure 2E, I, J) [28]. The *109-30-GAL4* driver is expressed only in stalk cells and in the stalk precursor cells in the germarium (Figure 2F, I, J) [29]. Unpaired GAL4 (*upd-GAL4*) drives expression exclusively in polar cells (Figure 2G and I) [30], a pair of FCs at the anterior and posterior end of the egg chamber that function as organizer cells (Figure 2J) [26]. Eyeless GAL4 (*ey-GAL4*) served as control and is not expressed in the ovaries (Figure 2H and I).

**Figure 3:**
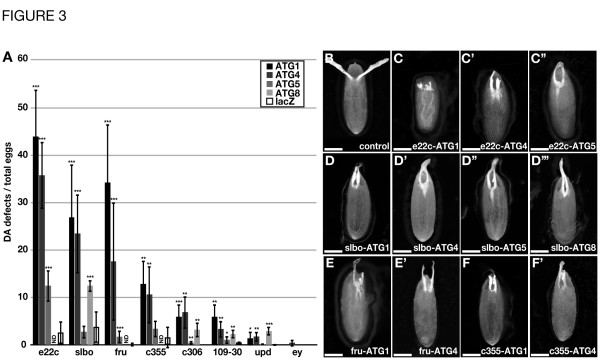
**Autophagy is important in certain FC subtypes for DA formation.** Spatially restricted GAL4 driver lines were used to express *ATG1*, *ATG4*, *ATG5* and *ATG8* RNAi and the effect of autophagy knock down on the dorsal appendage (DA) phenotype was quantified. Downregulation of *ATG* gene expression with the broad *e22c-GAL4* driver causes the most severe DA defects (**A** and **C**-**C”**). Knocking down *ATG* genes with *slbo-GAL4* and *fru-GAL4* causes similar strong phenotypes (**A** and **D**-**E’**). Overexpression of *ATG* RNAi with *c355-GAL4*, *c306-GAL4*, *109-30-GAL4* and *upd-GAL4* only generated a low percentage of eggs with defective DAs (**A** and **F**, **F’**). Similarly, the controls (*UAS>lacZ*, *ey-GAL4*) only occasionally showed defective eggs (**A** and **B**). A: Error bars show S.D. of the mean, *P*-values: * *P*<0.05, ** *P*<0.01, *** *P*<0.001. Eggs were collected from 5 females for each genotype, n=3. B-F’: Anterior is to the top, posterior to the bottom, dorsal to the front. Scale bar: 100 μm. Genotypes: GAL4 driver see Figure 2, *ATG*-RNAi lines see Additional file 2: Figure S2.

As the most persistent phenotype of eggs containing *ATG* mutant FC are malformed, shortened or missing dorsal appendages (DAs) (Figure 1G), we scored the frequency of this phenotype as a readout for the effect of *ATG* knock down in certain FC subpopulations [2]. The *e22c-GAL4* driver is expressed in virtually all FCs and as expected, this comprehensive expression pattern led to the most severe DA defects, resulting in 44% eggs with missing or malformed DAs after expression of *ATG1*-RNAi (Figure 3A and C). Also, expression of *ATG4*-RNAi (36%), and *ATG5*-RNAi (13%) with the *e22c-GAL4* driver resulted in significantly more eggs with DA defects when compared to control eggs (*lacZ*-RNAi, 3%) (Figure 3A and B, C’, C”). Expression of *ATG8*-RNAi led to pupal lethality probably due to expression of the *e22c-GAL4* driver in other tissues during development and the strength of the RNAi line used (Figure 3A). We obtained a slightly weaker DA phenotype by expression of *ATG1*-RNAi with *slbo-GAL4* (27%) or *fru-GAL4* (34%) (Figure 3A and D, E), and as for *e22c-GAL4*, the expression of *ATG4*-RNAi with *slbo-GAL4* (24%) and *fru-GAL4* (18%) was less severe than for *ATG1*-RNAi (Figure 3A and D’, E’). Expression of *ATG5*-RNAi with *slbo-GAL4* resulted in a minor number of defective eggs, however, the severity of DA defects was comparable to those obtained with the other *ATG*-RNAi lines (Figure 3D”).

Expression of *ATG8*-RNAi with *fru-GAL4* was lethal for the flies, but expression with *slbo-GAL4* led to significant DA defects (13%, Figure 3A). *c355-GAL4* driven expression of *ATG1*- and *ATG4*-RNAi still led to a defective DA rate of 13% and 11%, respectively (Figure 3A and F, F’), but expression of all other *ATG*-RNAi lines with the remaining GAL4 drivers (*c306-*, *109-30-*, and *upd-GAL4*) generally resulted in eggs with defective DAs in rates less than 7% (Figure 3A). In general, *ATG1*-RNAi caused the strongest phenotypes, followed by *ATG4*-, *ATG8*- and *ATG5*-RNAi. Expression of control *lacZ*-RNAi with the GAL4 lines only occasionally showed defective DAs (Figure 3A, white bars) and expression of *ATG*-RNAi using *ey-GAL4* as a control typically produced healthy eggs (Figure 2H, I, Figure 3A, B).

In order to examine the efficiency of *ATG* knockdown on the progression of autophagy, we used the FLP-out/GAL4 technique to induce clones expressing *ATG*-RNAi in the fat body (Additional file 2: Figure S2) [31]. The fat body of *Drosophila* rapidly reacts to starvation with the induction of autophagy, which can be easily monitored by lysotracker (LTR) staining [5]. Under fed conditions, LTR staining is diffuse, but accumulates in dots under starvation conditions (Additional file 2: Figure S2 A-B’). In fat body cell clones expressing the different RNAi lines, autophagy was inhibited as visualized by a strong reduction in LTR dots under starvation when compared to surrounding WT cells, indicating that the applied RNAi lines effectively knocked down *ATG* gene expression (Additional file 2: Figure S2 C-F’). Similarly, expression of *ATG*-RNAi also decreases LTR dot formation in FCs (Additional file 2: Figure S2 G- H’), validating the efficiency of the RNAi lines in downregulating autophagy in the ovaries.

In summary, inhibition of autophagy in follicular subgroups showed the strongest effect with the *e22c-*, *slbo-* and *fru-GAL4* driver. These three driver lines are expressed in border cells, posterior FCs and the anterior stretched and posterior columnar cells at later stages, but only some of the drivers are expressed in polar cells, stalk cells and the germarium. None or only minor DA defects were observed with *upd-GAL4*, which is exclusively expressed in polar cells, indicating that the polar cells are not responsible for the DA phenotypes seen with *e22c-*, *slbo-* and *fru-GAL4*. Further, expression solely in the stalk cells and stalk precursor cells in the germarium with the *109-30-GAL4* driver did not cause strong defects. Expression in border cells and the stretched and columnar cells is also driven by *c306-GAL4*, but the use of this driver only resulted in minor DA defects. This can be explained by the fact that *c306-GAL4*, although strongly expressed in stalk, border, and posterior cells in later stages, is only slightly expressed in terminal cells at earlier stages (Figure 2E). Thus, the differences in phenotypes caused by driver lines that show comparable expression patterns could thus be due to variations in expression levels or depend on the timing of expression. Expression in anterior stretched and posterior columnar cells as well as the border cells is also driven by *c355-GAL4* and indeed, *c355-GAL4* mediated expression of *ATG1* and *ATG4* RNAi resulted in DA defects.

Taken together, the posterior FCs cells are the FC subpopulation that shows a common expression pattern by *e22c*-, *slbo*-, and *fru-GAL4*. Furthermore, the anterior stretched and posterior columnar cells at later stages also show a common expression pattern with the three mentioned drivers and *c355-GAL4*, and the expression of *ATG*-RNAi with *c355-Gal4* also resulted in considerable DA defects. Thus, these FC subpopulations are likely to be involved in generating the DA defect.

Malformations of DAs represent the most persistent phenotype detected in late stage autophagy deficient eggs, which could be explained by the lack of autophagy in anterior dorsal FCs. We also observed oogenesis defects in early stages, however, since these phenotypes were more difficult to score and less stringent, we were unable to designate FC subpopulations responsible for these defects. Thus, FC subtypes other than the anterior dorsal FCs may also be sensitive to the lack of autophagy to affect oogenesis. Interestingly, many of the phenotypes observed in *ATG1* mutant eggs resembled those described in mutants of the classical signaling pathways that control oogenesis [21].

### Lack of autophagy alters Notch signaling at specific stages during *Drosophila* oogenesis

Signaling between the oocyte and the somatic FCs determines the body axes during *Drosophila* oogenesis. The discrete patterning of the FCs along this axis mediated by JAK/STAT, Delta-Notch and EGFR signaling, is important for the establishment of anterior-posterior polarity [13,32]. We show that the lack of autophagy in FCs disturbs egg development and leads to severe DA defects. All three pathways are active in subsets of FCs, however, we do not observe DA defects by knocking down *ATG* genes in polar cells (Figure 3), suggesting that the loss of autophagy in cells requiring JAK/STAT signaling does not affect egg development. Further, we recently demonstrated that the necessity of autophagy in FCs depends on a cellular context where DA defects are only seen in eggs with *ATG* mutant FCs and WT GCs [2]. Since secretion of upd and activation of JAK/STAT signaling in neighbouring FCs is a GC independent signaling process, we exclude autophagy dependent modulation of JAK/STAT from causing DA defects [33].

EGFR signaling is activated in posterior FCs upon Gurken translation by the oocyte, and movement of the oocyte nucleus to a lateral-anterior position requires an unknown back-signaling by the FCs [32,34]. Thus, it could be possible that autophagy deficient FCs are impaired in transmitting the signal back to the oocyte. However, we did not detect any abnormalities in the accumulation of Gurken protein within the oocyte or Gurken uptake by *ATG1* mutant FCs (Additional file 3: Figure S3 A-B”) nor in the activation of the EGFR downstream targets Broad and Kekkon (Additional file 3: Figure S3 C-F”). Moreover, we would expect to see the same phenotype in a situation where both FCs and GCs are mutant, since autophagy deficiency in the GCs would not be able to further modify the deregulated back-signaling. Consequently, we conclude that EGFR signaling is not altered by the lack of autophagy in FCs.

**Figure 4:**
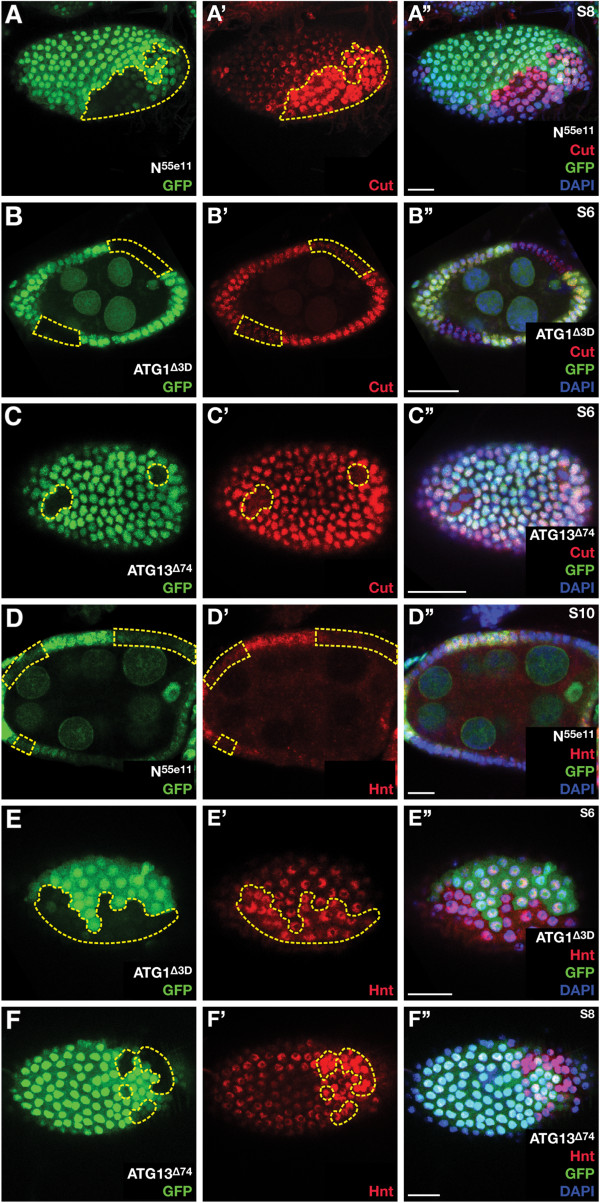
**Autophagy modulates Notch signaling in *Drosophila *FCs.** Notch signaling activity in eggs containing *ATG* mutant FC clones was monitored using the downstream targets Cut and Hindsight (Hnt). (**A**) In HS-FLP induced FC clones mutant for *Notch* (marked by the lack of GFP, outlined in yellow), Cut is not downregulated post stage 6. In contrast, *ATG1* (**B**) or *ATG13* (**C**) mutant FCs show a precocious downregulation of Cut compared to WT cells. (**D**) Under normal conditions, the expression of Hnt is upregulated by Notch signaling, which is not accomplished in cells mutant for *Notch* (marked by the lack of GFP, outlined in yellow). In FC clones mutant for *ATG1* (**E**) or *ATG13* (**F**), Hnt upregulation occurs earlier or stronger than in surrounding WT cells. Anterior is to the left, posterior to the right. Scale bar: 20 μm. Genotypes: A, D: *hs flp FRT19A-UbiGFP/N*^*55e11*^*FRT19A*. B, E: *hs flp/+; ATG1*^*∆ 3D*^*FRT80B/FRT80B-UbiGFP*. C, F: *hs flp/+; ATG13*^*∆ 74*^*FRT82/FRT82-UbiGFP* .

To determine whether Notch signaling is affected in autophagy deficient FCs, we monitored the expression of two Notch downstream target genes, *Cut* and *Hindsight* (*Hnt*). The transcription factor Cut is expressed during oogenesis in all FCs until stage 6. Concomitant with the cell-cycle switch, Notch pathway activation downregulates *Cut* expression and Cut protein vanishes (Additional file 4: Figure S4 A, B) [35]. Subsequently, Notch signaling leads to expression of another transcription factor, Hnt, which can be detected in FCs from stage 6 onwards (Additional file 4: Figure S4 A, C) [36]. In FCs mutant for *Notch*, Cut is not downregulated and remains expressed beyond stage 6 (Figure 4A-A”). Conversely, *ATG1* and *ATG13* mutant FCs at stage 6 display weaker Cut staining compared to surrounding WT cells (Figure 4B-C”). However, the modulation of Cut expression is only seen in stage 6/7 egg chambers, and FC clones in egg chambers of earlier stages displayed Cut stainings comparable to surrounding WT cells (data not shown), suggesting an earlier downregulation of Cut in autophagy deficient cells rather than a complete inhibition. On the other hand, Notch mutant FCs fail to upregulate Hnt after the cell cycle switch (Figure 4D-D”). However, *ATG1* and *ATG13* mutant FC clones display precocious or stronger upregulation of Hnt (Figure 4E-F”) compared to WT FCs. Thus, *ATG* deficient FC clones display the opposite phenotype of FC clones mutant for Notch, suggesting that Notch signaling might be enhanced by the lack of autophagy. Indeed, the overexpression of a functional Notch-GFP construct also displayed enhanced Hnt staining (data not shown). Notably, the phenotypes observed in autophagy deficient eggs resemble those described for mutants in Notch signaling components. Using a temperature sensitive allele of *Notch* (*N*^*ts1*^), Xu et al. observed fused egg chambers with a high frequency, similarly to eggs containing *ATG1* mutant FC clones (Figure 1). Furthermore, the *N*^*ts1*^ allele is associated with malformed DAs [37]. In addition, downregulating the expression of fringe, a protein that modulates the ability of Serrate and Delta to activate Notch, resulted in malformed egg chambers and defective DAs [38].

Interestingly, autophagy deficiency only affects Notch signaling in a cellular context when FCs are mutant and the germline is WT, since eggs lacking *ATG1* function in both GCs and FCs do not show precocious activation of the Notch pathway (Additional file 4: Figure S4 D-D”). This is in accordance with our incompatibility model where dysfunctional signaling between germline and FCs is responsible for the oogenesis defect. We thus propose that the lack of autophagy in the germline may reduce the amount of active ligand to counteract the increased receptor signaling in the autophagy-deficient FCs.

## Discussion

Our previous findings indicated that autophagy is especially required in FCs during oogenesis [2]. Here, we demonstrate that autophagy deficiency in the FCs causes severe egg chamber defects, and that autophagy is presumably required in the posterior FCs, in columnar cells and in the anterior stretched cells. FCs are important for patterning of the egg, and functional cell-cell signaling is crucial for egg development [13]. We formerly showed that autophagy deficiency only affects oogenesis in a cellular context in which FCs are mutant for *ATG* genes and GCs are WT, hypothesizing that autophagy could be implicated in the regulation of signal transduction pathways required for oogenesis [2]. In fact, our present results demonstrate that defective autophagy leads to the modulation of Notch downstream effectors. This finding may be especially relevant since dysregulation of Notch has been implicated in tumorigenesis [39].

Interestingly, generation of *ATG* deficient FCs leads to a wide range of phenotypes, many of which are observed in mutants of signaling pathways needed for egg differentiation: Delta-Notch, JAK/STAT and EGFR. For example, egg chambers containing *Notch* mutant FC clones lack stalk cells and display encapsulation defects, resulting in compound egg chambers with increased numbers of cysts [17]. Moreover, fused egg chambers and oocyte mislocalization are also observed in mutants of the JAK/STAT and EGFR pathway [40,41].

Using GAL4-driver specific for subsets of FCs, we showed that autophagy deficiency in posterior FCs, columnar cells and in anterior stretched FCs leads to severe defects in DA formation and thus non-functional eggs. The three classical signaling pathways that control oogenesis- Notch, JAK/STAT and EGFR signaling- are active in various subsets of FCs at different stages of egg development. We recently demonstrated that the requirement of autophagy depends on the cellular context, since DA defects are only seen in eggs with *ATG* mutant FCs and WT GCs [2]. Because secretion of upd and activation of JAK/STAT signaling in neighbouring FCs is a GC independent signaling process [33], and since we did not observe DA defects by *ATG*-RNAi expression in polar cells, we exclude autophagy dependent modulation of JAK/STAT from causing DA defects. EGFR signaling is activated in posterior FCs, and the movement of the oocyte nucleus to a lateral-anterior position requires an unknown back-signaling by the FCs [32,34]. The transmission of this signal could be impaired in autophagy deficient FCs. However, egg chambers with *ATG* deficient FCs displayed normal Gurken signaling and movement of the oocyte to the designated places. Consequently, we also exclude defective EGFR signaling from causing the observed DA defects. Interestingly, the lack of the cysteine protease ATG4 was shown to enhance the *Notch* mutant wing phenotype in *Drosophila*, thus, it is tempting to speculate that impaired autophagy may also lead to dysregulation of Notch in the ovaries [42]. Using two readouts for Notch activity, Cut and Hnt, we showed that loss of autophagy in *ATG1* and *ATG13* mutant FC clones modulates the expression of these Notch targets. This modulation is only visible in stage 6 of oogenesis when the Notch pathway is switched on by the expression of Delta in the germline, suggesting that Notch deregulation caused by the lack of autophagy can be rapidly compensated in later stages. It has been shown that endocytosis and endocytic trafficking regulate receptor activity, and that retention of Notch in endosomal vesicles accelerates its intramembrane cleavage and intensifies Notch signaling [43]. Recently, the *Drosophila UV-resistance associated gene* (*UVRAG*), which is implicated in autophagy and endocytosis, was shown to regulate Notch receptor endocytosis and subsequent degradation [44]. The authors show that *UVRAG* mutant cells are impaired in activating autophagy, but assume that defects in endocytosis are responsible for Notch deregulation. However, the authors also suggest that UVRAG is required for targeting of Notch to lysosomes [44]. Furthermore, loss of the phosphatidylinositol 3-kinase Vps34, which is required for autophagy and endocytosis, results in the accumulation of Notch [45]. It is feasible that the strong phenotype observed in *UVRAG* and *Vps34* mutants is a combination of deregulated endocytic trafficking and autophagosomal receptor degradation, whereas the sole loss of autophagy has only a minor or temporary impact on degradation and can rapidly be compensated by other mechanism, e.g. direct fusion of endosomes with lysosomes without the involvement of the autophagic machinery. In addition, *ESCRT* mutants show a ligand independent activation of Notch signaling, which might result from altered trafficking and endosomal accumulation, and ESCRT is also required for autophagy [43,46]. Thus, several proteins are implicated in both autophagy and endosomal receptor sorting, and intersections between the endosomal and autophagic pathways have been described [47,48]. In *ATG1* mutant FCs, Cut and Hnt expression is inversely regulated compared to *Notch* loss-of-function clones, which suggests an activation of Notch signaling. This is in accordance with *UVRAG*, *Vps34* or *ESCRT* mutants, where Notch signaling is also increased [43-45]. Endocytic internalization and trafficking is essential for the cleavage and release of the Notch intracellular domain (NICD), which translocates to the nucleus to activate the transcription of target genes [43]. In fact, mutants that increase endosomal retention of the Notch receptor, e.g. *ESCRT* mutants, show enhanced Notch activity [43]. We propose that the absence of autophagy might lead to a pause in the normally rapid endosomal processing of internalized Notch, which in turn leads to pronounced NICD cleavage and enhanced Notch activity. However, compound egg chambers and the lack of stalk cells are phenotypes known for *Notch* mutants, whereas constitutive active Notch signaling leads to longer stalk cells [49], a phenotype that is not observed in ovaries with *ATG* mutant FCs. Nevertheless, since modulation of Notch signaling is only observed in stage 6 egg chambers, it is possible that either this dysregulation is not strong enough to cause severe gain of function Notch phenotypes, or that autophagy has no impact on Notch signaling during the differentiation of stalk cells in early oogenesis. Although we did not observe modulations in EGFR signaling, the possibility remains that autophagy has stage- or cell type specific functions in the modulation of other cell-cell signaling pathways that could cause the observed egg chamber defects. The EGF receptor is also regulated by endocytosis and endosomal trafficking [50], thus, autophagy might be involved in EGFR receptor degradation as well.

Autophagy has recently been linked to the proteasomal pathway and serves to selectively degrade ubiquitinated proteins [51]. Carrier proteins, such as the multi-binding domain protein p62 (Ref(2)P in *Drosophila*), bind ubiquitin and LC3 (ATG8 in *Drosophila*) to target proteins to autophagosomes, as shown for the Wnt-signaling protein Dishevelled [52]. Interestingly, FC mutants for the SCF protein *Slimb*, a E3 ubiquitin ligase complex component, also lack stalk cells and show dorsal appendage (DA) defects [53], and SCF complex family members are implicated in targeting the Notch receptor for degradation [54]. This could hint to a common mechanism of Notch degradation failure leading to DA defects. Another E3 ubiquitin ligase, c-Cbl, was recently shown to mediate autophagic targeting and selective degradation of the tyrosin kinase Src through direct binding to LC3 [55]. Given that D-Cbl, the only *Drosophila* E3 ubiquitin ligase of the Cbl family, negatively regulates Notch activity, the binding of D-Cbl to ATG8 could target Notch to autophagosomes for degradation [56]. Multiple mechanisms to degrade NICD might be important to decrease Notch signaling since the sole inhibition of Delta binding to Notch does not block NICD activity [57].

Notch is activated in FCs by a signal from the germline, and both receptor and ligand are regulated by internalization and endosomal trafficking [58]. Interestingly, mutants defective in endocytosis show abnormal trafficking of Delta and reduced Notch signaling [59]. Moreover, mono-ubiquitination of Delta is required for endocytosis and receptor activation [60]. Thus, in a situation where both FCs and GCs are *ATG* deficient, the lack of autophagy may modulate endocytic processing of Delta in the germline, leading to reduced ligand signaling that is able to compensate for the increased activity in autophagy deficient FCs. Indeed, it was shown that liquid facets (lqf), the *Drosophila* homologue of Epsin that is required for Delta endocytosis, is also implicated in autophagy [61,62].

## Conclusions

In summary, our work shows that autophagy is critical in *Drosophila* FCs and has the ability to modulate the expression of Notch downstream targets. Since Notch signaling plays important roles in tissue differentiation and tumorigenesis, this alternative way of endosomal receptor regulation might be relevant for studies concerning cancer treatment. Notably, the situation in a tumor resembles our experimental set up in which an imbalance between WT and mutant tissue assigns a fate to a certain cell type. Thus, the dysregulation of autophagy may represent an advantage to promote carcinogenesis.

Autophagy and endocytosis equally represent relevant inputs for lysosomal degradation, but the interplay of both pathways is still poorly understood. Further studies will be required to clarify whether autophagy is indeed involved in the endocytic regulation of ligands and receptors in cell signaling pathways.

## Methods

### *Drosophila* maintenance and stocks

Flies were raised on standard yeast/cornmeal agar at 25°C. *Drosophila melanogaster* stocks used: *ATG1*^*∆ 3D*^*FRT80B*, *ATG5-RNAi*, *ATG13*^*∆ 74*^*FRT82* (kindly provided by T. Neufeld) [5,63]. *ATG1*^*∆ 3D*^*FRT80B-UbiGFP* (recombined from *ATG1*^*∆ 3D*^, T.N.). *UAS-Notch-GFP* (kindly provided by S. Hayashi) [64]*. ATG1-RNAi* (GD16133), *ATG4-RNAi* (KK107317), *ATG8-RNAi* (KK109654), *lacZ-RNAi*, (VDRC, Vienna, Austria). *e22c-GAL4 UAS>FLP;FRT80-UbiGFP*, *e22c-GAL4 UAS>FLP;FRT82-UbiGFP*, *P[w+ lac-Z]BB142* (=kekkon-lacZ) (kindly provided by T. Schüpbach) [25,65,66]. *fru-GAL4* (*168-GAL4*) (kindly provided by A.-M. Pret). *upd-GAL4* (kindly provided by S. Noselli) [30]. *c306-GAL4* (3743) [28], *c355-GAL4* (3750) [28], *109-30-GAL4* (7023) [29], *slbo-GAL4* (6458), *ey-GAL4*, *UAS-GFP, N*^*55e11*^*FRT19A* (28813), *FRT19-UbiGFP*, *FRT80B-UbiGFP*, *FRT82-UbiGFP*, *FRT80iso*, *FRT82iso*, *FRT80 w+*, *y w* (Bloomington *Drosophila* Stock Center, Indiana University, IN, USA). *Ovo*^*D*^*-FRT80* (kindly provided by P. Gallant/P. Rorth).

### LTR assay, starvation, tissue preparation, immunostainings and microscopy

For LTR assays, early L3 larvae were starved for 2h in 10% sucrose in PBS solution. Fat body tissue was dissected in PBS, incubated for 1 min in 100 mM Lysotracker red DND-99 (Invitrogen, Molecular Probes, Basel, Switzerland) to label acidic organelles including autolysosomes, washed three times in PBS and live imaged using a confocal microscope (see below). Ovaries were dissected in PBS, fixed in 4% paraformaldehyde (PFA) for 20 min, embedded in mounting medium with DAPI (Vectashield, Vector Laboratories, Inc., Burlingame, CA, USA). Ovaries for immunostainings were prepared as described elsewhere [2], except immunostainings with β-Galactosidase antibodies (lacZ stainings), which were prepared without Methanol dehydration. Primary antibodies used: mouse anti-β-Galactosidase (1:300) (Z378A, Promega, WI, USA), mouse anti-Gurken (1D12) (1:50), mouse anti-Broad-core (25E9.D7) (1:100), mouse anti-Hnt (1:100), mouse anti-Cut (1:100) (Developmental Studies Hybridoma Bank, IA, USA). Secondary antibody: Cy3 anti-mouse (1:300) (GE Healthcare, Germany). Images were obtained using a confocal microscope (Leica, Wetzlar, Germany, DM5500Q, TCS-SPE; objective lenses: Leica, 20x (0.70), 40x (1.15), 63x (1.30); acquisition software: LAS AF v.2.0.1, Leica, Wetzlar, Germany) and a digital microscope (Keyence, Osaka, Japan, VHX-1000D; objective lens: VH-Z100R 100x-1000x zoom lens) at room temperature and edited using Adobe Illustrator and Photoshop CS5.

### Generation of mosaic tissues

The FLP/FRT recombination method was used to generate FC, germline and fatbody clones. Heat-shock induced FC clones mutant for *ATG1*, *ATG13*, or *Notch* were generated by placing the flies of the genotypes *FRT80B-ATG1*^*∆ 3D*^*/FRT80B-UbiGFP*, *FRT82-ATG13*^*∆ 74*^*/FRT82-UbiGFP* or *FRT19A-N*^*55e11*^*/FRT19A-UbiGFP* for 1 h at 37°C during larval development on day 2, 3 and 4 after egg laying. For *e22c-GAL4 UAS>FLP* induced clones, flies were crossed with *FRT80B-ATG1*^*∆ 3D*^*or FRT82-ATG13*^*∆ 74*^ and dissected 4 days after hatching. The frequency of clones induced using this method has been described previously [2]. *Ovo*^*D*^ clones were induced by heat shock (HS) as described elsewhere [12]. Fat body FLP out clones were achieved though HS independent induction as described elsewhere [67].

### Egg laying analysis and quantification of DA defects

For egg laying analysis, females with the appropriate genotype were mated with WT males in single vials and eggs with intact and defective DAs (shortened, missing or malformed) were quantified every day for 4 consecutive days. For each genotype and independent experiment (n=3), the eggs of 5 individual females were counted. *P*-values were calculated with t-test (two tailed, two-samples unequal variance) using Excel, the comparison was to the control (*lacZ*).

## Abbreviations

ATG: Autophagy related genes; DA: Dorsal appendage; Dl: Delta; EGFR: Epidermal growth factor receptor; ESCRT: Endosomal sorting complex required for transport; ey: Eyeless; FC: Follicle cell; FLP-FRT: Flipase recognition target; fru: Fruitless; GC: Germ cell; GLC: Germline clone; Hnt: Hindsight; HS: Heat shock; lqf: Liquid facets; LTR: Lysotracker; NC: Nurse cell; NICD: Notch intracellular domain; PFA: Paraformaldehyde; PNCN: Persisting nurse cell nuclei; RNAi: Interfering ribonucleic acid; SCF: Skp, Cullin, F-box complex; S.D: Standard deviation; slbo: Slow border cell; upd: Unpaired; UVRAG: UV-resistance associated gene; WT: Wild type.

## Competing interests

The authors declare that they have no competing financial, professional or personal interests that might have influenced the performance or presentation of the work described in this manuscript.

## Authors’ contributions

JB carried out the experiments, participated in the design and drafted the manuscript. EH participated in the design of the study and in revising the manuscript. KK conceived of the study, participated in its design and organization and helped to draft the manuscript. All authors read and approved the final manuscript.

## Supplementary Material

Additional file 1 Figure S1 Generation of *ATG* mutant FC clones by different techniques. (A-B’) Producing germline clones (GLCs) using the HS-FLP FRT *Ovo*^*D*^ technique induces a complete mutant germline since GCs homozygous for the dominant female sterile mutation *Ovo*^*D*^ die. However, mutant clones are also induced in the somatic tissue where the mutation is not lethal. Thus, eggs with a mosaic FC epithelium occur and develop (A-B’, arrowheads, marked by the lack of GFP). (C-D’) For comparison, HS-FLP induced FC clones (*ATG1* mutant clones are marked with GFP) (C, C’) and *e22c-GAL4, UAS>FLP* induced *ATG1* FC clones (mutant clones are marked by the lack of GFP) are shown (D, D’). Anterior is to the left, posterior to the right. Scale bar: 50 μm. Genotypes: A: *hs flp/+; Ovo*^*D*^*FRT80B/FRT80B-UbiGFP*, B: *hs flp/+; Ovo*^*D*^*FRT80B/ATG1*^*∆ 3D*^*FRT80B-UbiGFP*, C: *hs flp/+; w*^*+*^*FRT80B/ATG1*^*∆ 3D*^*FRT80B-UbiGFP*, D: *hs flp/+; e22c UAS>FLP; FRT80B-UbiGFP/ATG1*^*∆ 3D*^*FRT80B-UbiGFP*. Click here for file

Additional file 2 Figure S2 Autophagic activity is reduced in *ATG*-RNAi treated cells. LTR staining of FLP-out/GAL4 induced fat body and FC clones expressing *ATG*-RNAi. (A, A’) Under well-fed conditions, LTR staining is ubiquitously distributed in wild type (WT) cells and cells expressing control *lacZ*-RNAi (marked with GFP). (B, B’) Under starvation, control *lacZ*-RNAi expressing cells accumulate LTR positive dots as in surrounding WT cells. (C-F’) Expression of *ATG1*-RNAi (cells marked with GFP) inhibits the formation of LTR positive dots compared to surrounding WT cells (C, C’). The same is seen for *ATG4*-RNAi (D, D’), *ATG5*-RNAi (E, E’) and *ATG8*-RNAi (F, F’). Expression of *ATG*-RNAi equally inhibits LTR dot formation in FCs using *ATG1-RNAi* (G, G’) or *ATG8-RNAi* (H, H’). Scale Bar: 50 μm (A-F), 25 μm (G-H). Genotypes: A, B: *hs flp/UAS>lacZ*^*RNAi*^*;;act>CD2>GAL4 UAS>GFPnls/+*, C, G: *hs flp/+;UAS>ATG1*^*RNAi*^*/+;act>CD2>GAL4 UAS>GFPnls/+*, D: *hs flp/+;UAS>ATG4*^*RNAi*^*/+;act>CD2>GAL4 UAS>GFPnls/+*, E: *hs flp/UAS>ATG5*^*RNAi*^*;;act>CD2>GAL4 UAS>GFPnls/+*, F, H: *hs flp/+;UAS>ATG8*^*RNAi*^*/+;act>CD2>GAL4 UAS>GFPnls/+*. Click here for file

Additional file 3 Figure S3 Lack of autophagy does not affect EGFR signaling activity. HS-FLP induced *ATG1* clones (marked by the lack of GFP) were examined for different read-outs of EGFR signaling. (A, B) Gurken (Grk) protein (stained in red) is translated by the oocyte and activates the EGF receptor in adjacent FCs. Normal accumulation of Grk in the posterior corner of the oocyte (arrowhead) and uptake of Grk by FCs (arrows) are seen in stage 7 egg chambers (A-A”), as well as after the movement of the nucleus to the anterior-dorsal side in stage 10 (B-B”). (C, D) The transcription factor Broad is expressed in all stage 9 oocyte-associated FCs and no difference is seen between WT and *ATG1* mutant FCs (outlined in yellow) (C-C”). By stage 10, Broad gets repressed in midline FCs and all other FCs except the two patches of future roof cells, which is equally seen in WT and *ATG1* mutant FCs (outlined in yellow) (D-D”). (E, F) In eggs containing *ATG1* mutant FCs, a normal distribution of kekkon (kek, stained in red) expression is seen in FCs overlying the nucleus in stage 10 eggs (E-E”) and also in stage 11 eggs in cells that later form the dorsal appendages (F-F”). Anterior is to the left, posterior to the right. In B and E, dorsal is to the top. Scale bar: 50 μm. Genotypes: A-D: *ATG1*^*∆ 3D*^*-FRT80B*/*FRT80-UbiGFP*. E, F: *P[w+ lac-Z]BB142* (=kekkon-lacZ); *ATG1*^*∆ 3D*^*-FRT80B*/*FRT80-UbiGFP*. Click here for file

Additional file 4 Figure S4 Expression pattern of the Notch signaling targets Cut and Hnt. (A) Schematic representation of Notch signaling activity. Until stage 5, Delta (Dl) is not expressed by the germline, Notch is not activated in FCs, and Cut is expressed whereas Hnt is absent. By stage 6, Dl is expressed by the germline and activates Notch in FCs, Cut is downregulated, and Hnt is expressed (B) Expression of Cut starting in the germarium and continuing until stage 6. (C) Expression of Hnt is absent in early stages but expression is activated by stage 6. (D) Eggs lacking ATG1 function in both GCs and FCs (marked by the lack of GFP) show normal activation of the Notch pathway with Cut expression levels comparable to WT eggs. Anterior is to the left, posterior to the right. Scale bar: 50 μm (B-C), 20 μm (D). Genotypes: B, C: *y w*. D: *hs flp/+; ATG1*^*∆ 3D*^*FRT80B/FRT80B-UbiGFP*. Click here for file
