# Supplementary figures and images for: The lack of autophagy triggers precocious activation of Notch signaling during Drosophila oogenesis
Source: BMC Dev Biol. 2012 Dec 5;12:35. doi: 10.1186/1471-213X-12-35 (PMC3564699; doi:10.1186/1471-213X-12-35)

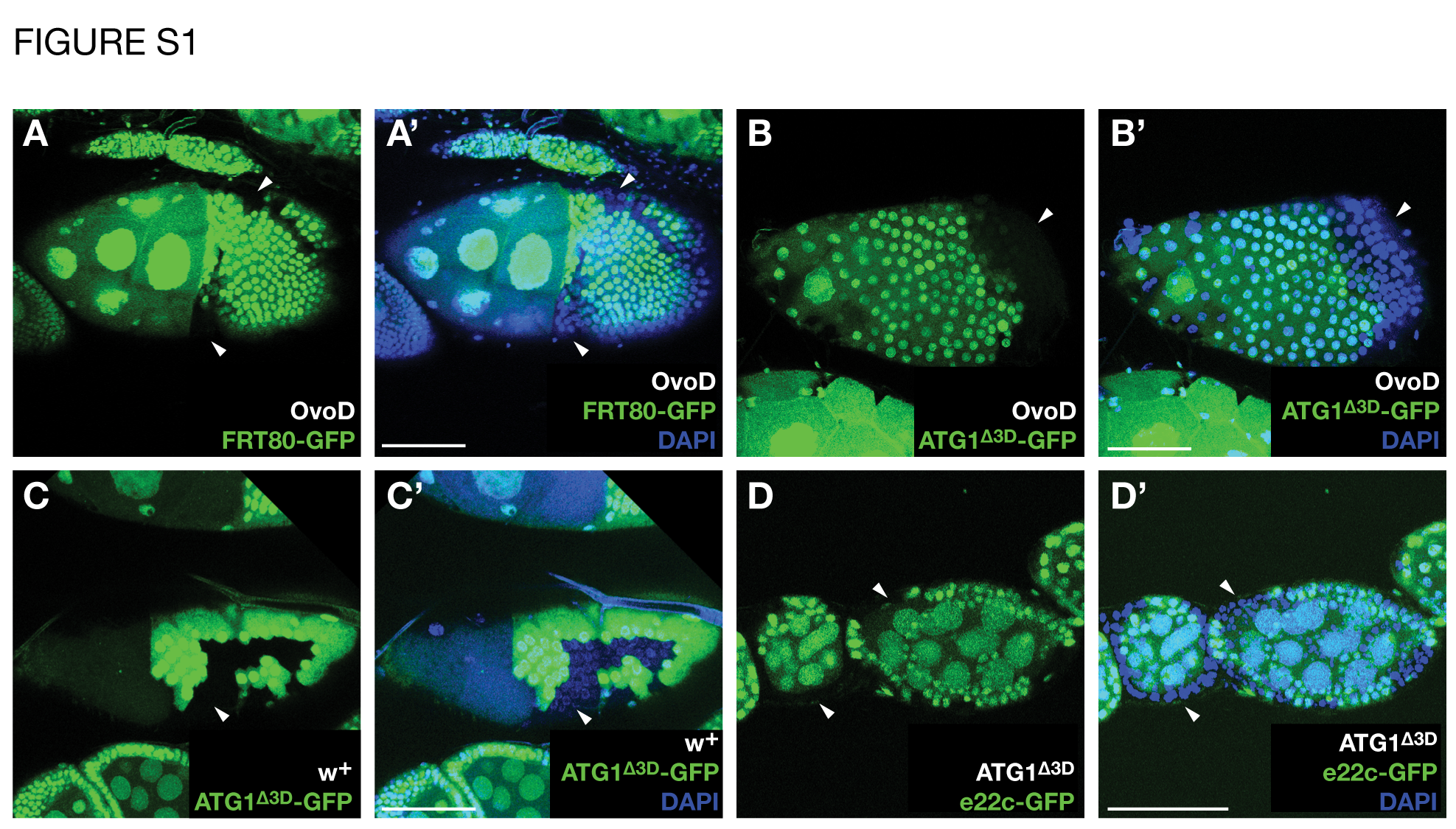

Supplement: Additional file 1 Figure S1 — Generation of ATG mutant FC clones by different techniques. (A-B’) Producing germline clones (GLCs) using the HS-FLP FRT OvoD technique induces a complete mutant germline since GCs homozygous for the dominant female sterile mutation OvoD die. However, mutant clones are also induced in the somatic tissue where the mutation is not lethal. Thus, eggs with a mosaic FC epithelium occur and develop (A-B’, arrowheads, marked by the lack of GFP). (C-D’) For comparison, HS-FLP induced FC clones (ATG1 mutant clones are marked with GFP) (C, C’) and e22c-GAL4, UAS>FLP induced ATG1 FC clones (mutant clones are marked by the lack of GFP) are shown (D, D’). Anterior is to the left, posterior to the right. Scale bar: 50 μm. Genotypes: A: hs flp/+; OvoDFRT80B/FRT80B-UbiGFP, B: hs flp/+; OvoDFRT80B/ATG1∆ 3DFRT80B-UbiGFP, C: hs flp/+; w+FRT80B/ATG1∆ 3DFRT80B-UbiGFP, D: hs flp/+; e22c UAS>FLP; FRT80B-UbiGFP/ATG1∆ 3DFRT80B-UbiGFP. [file 1471-213X-12-35-S1.tiff]

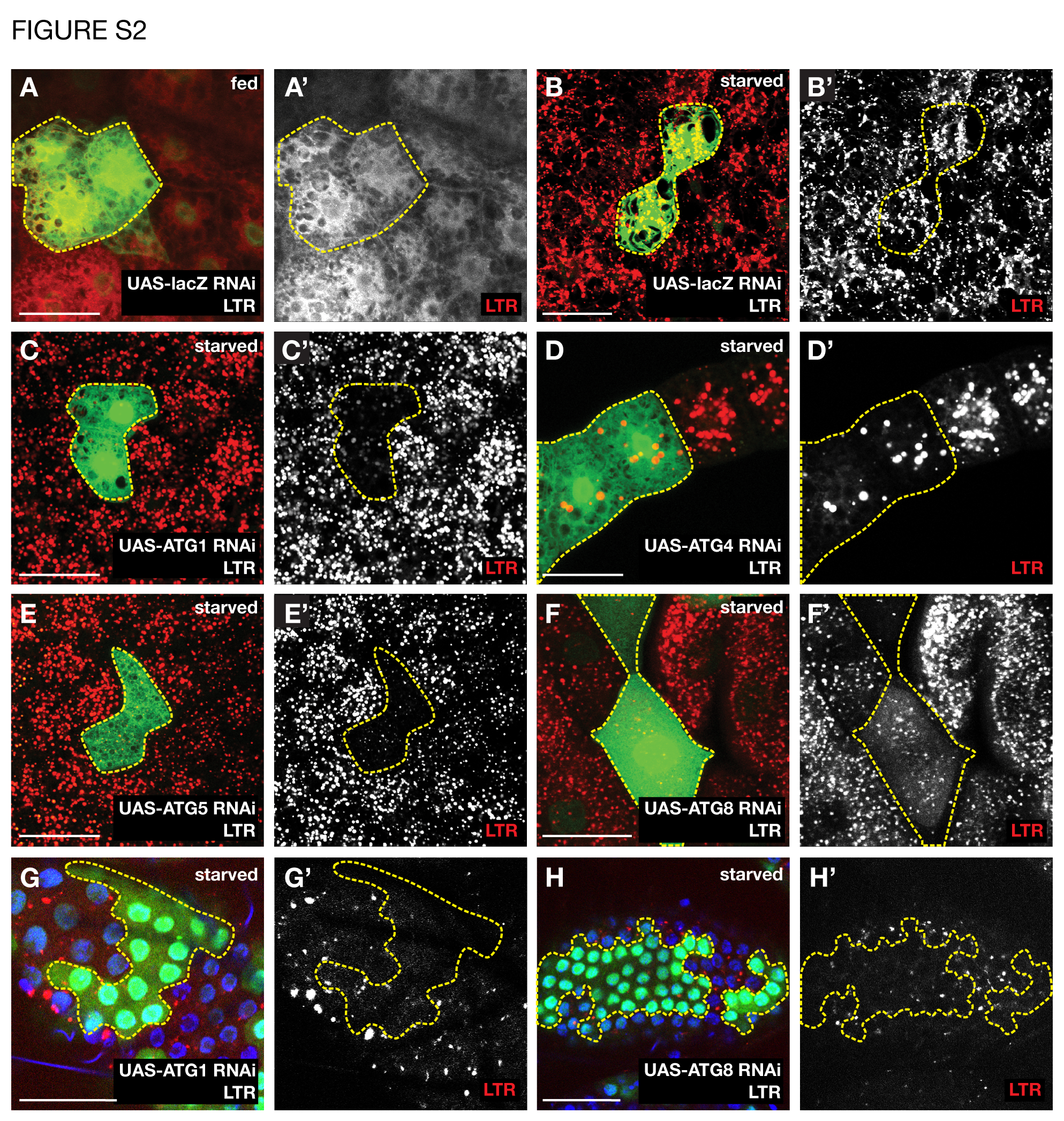

Supplement: Additional file 2 Figure S2 — Autophagic activity is reduced in ATG-RNAi treated cells. LTR staining of FLP-out/GAL4 induced fat body and FC clones expressing ATG-RNAi. (A, A’) Under well-fed conditions, LTR staining is ubiquitously distributed in wild type (WT) cells and cells expressing control lacZ-RNAi (marked with GFP). (B, B’) Under starvation, control lacZ-RNAi expressing cells accumulate LTR positive dots as in surrounding WT cells. (C-F’) Expression of ATG1-RNAi (cells marked with GFP) inhibits the formation of LTR positive dots compared to surrounding WT cells (C, C’). The same is seen for ATG4-RNAi (D, D’), ATG5-RNAi (E, E’) and ATG8-RNAi (F, F’). Expression of ATG-RNAi equally inhibits LTR dot formation in FCs using ATG1-RNAi (G, G’) or ATG8-RNAi (H, H’). Scale Bar: 50 μm (A-F), 25 μm (G-H). Genotypes: A, B: hs flp/UAS>lacZRNAi;;act>CD2>GAL4 UAS>GFPnls/+, C, G: hs flp/+;UAS>ATG1RNAi/+;act>CD2>GAL4 UAS>GFPnls/+, D: hs flp/+;UAS>ATG4RNAi/+;act>CD2>GAL4 UAS>GFPnls/+, E: hs flp/UAS>ATG5RNAi;;act>CD2>GAL4 UAS>GFPnls/+, F, H: hs flp/+;UAS>ATG8RNAi/+;act>CD2>GAL4 UAS>GFPnls/+. [file 1471-213X-12-35-S2.tiff]

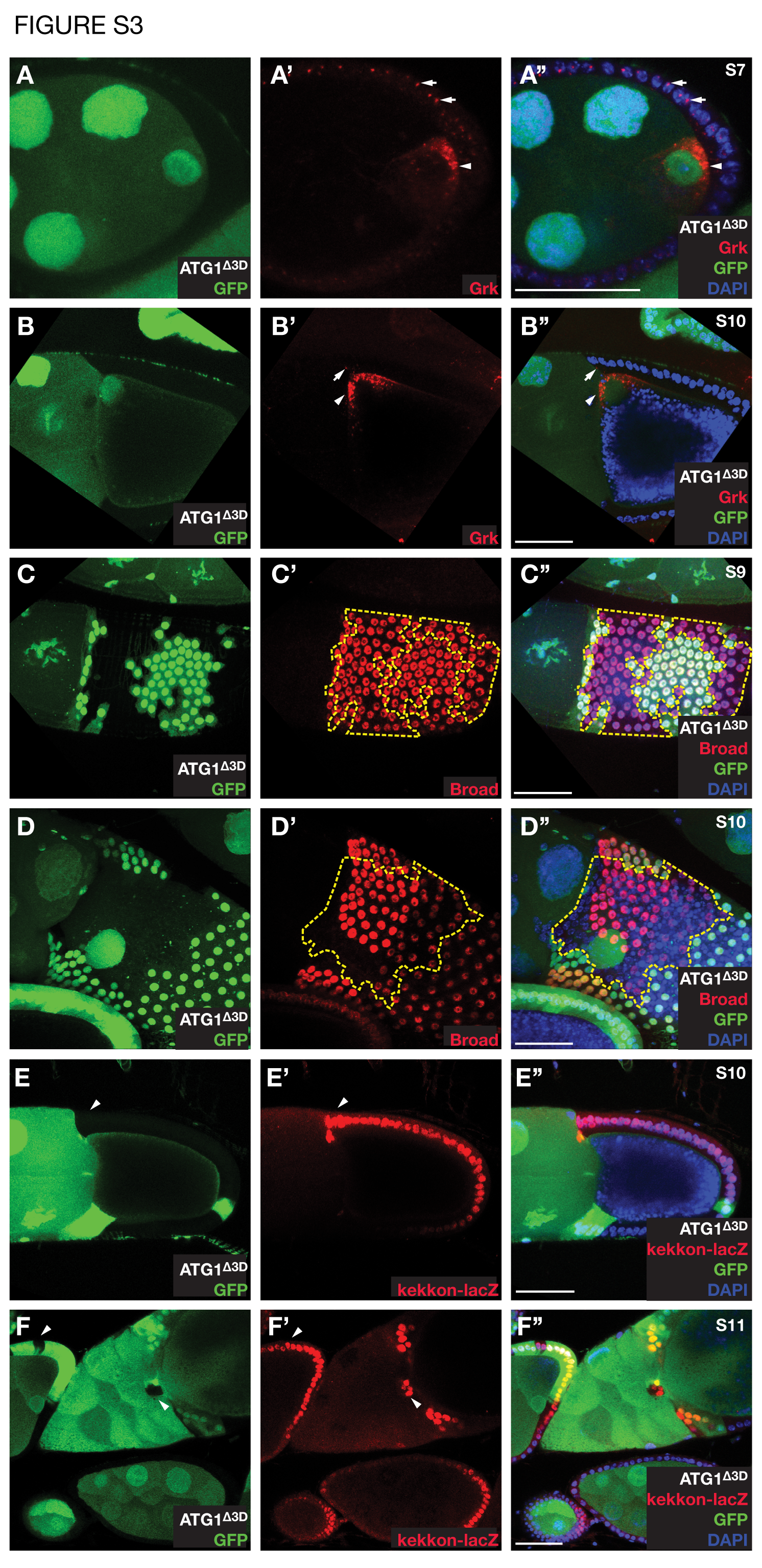

Supplement: Additional file 3 Figure S3 — Lack of autophagy does not affect EGFR signaling activity. HS-FLP induced ATG1 clones (marked by the lack of GFP) were examined for different read-outs of EGFR signaling. (A, B) Gurken (Grk) protein (stained in red) is translated by the oocyte and activates the EGF receptor in adjacent FCs. Normal accumulation of Grk in the posterior corner of the oocyte (arrowhead) and uptake of Grk by FCs (arrows) are seen in stage 7 egg chambers (A-A”), as well as after the movement of the nucleus to the anterior-dorsal side in stage 10 (B-B”). (C, D) The transcription factor Broad is expressed in all stage 9 oocyte-associated FCs and no difference is seen between WT and ATG1 mutant FCs (outlined in yellow) (C-C”). By stage 10, Broad gets repressed in midline FCs and all other FCs except the two patches of future roof cells, which is equally seen in WT and ATG1 mutant FCs (outlined in yellow) (D-D”). (E, F) In eggs containing ATG1 mutant FCs, a normal distribution of kekkon (kek, stained in red) expression is seen in FCs overlying the nucleus in stage 10 eggs (E-E”) and also in stage 11 eggs in cells that later form the dorsal appendages (F-F”). Anterior is to the left, posterior to the right. In B and E, dorsal is to the top. Scale bar: 50 μm. Genotypes: A-D: ATG1∆ 3D-FRT80B/FRT80-UbiGFP. E, F: P[w+ lac-Z]BB142 (=kekkon-lacZ); ATG1∆ 3D-FRT80B/FRT80-UbiGFP. [file 1471-213X-12-35-S3.tiff]

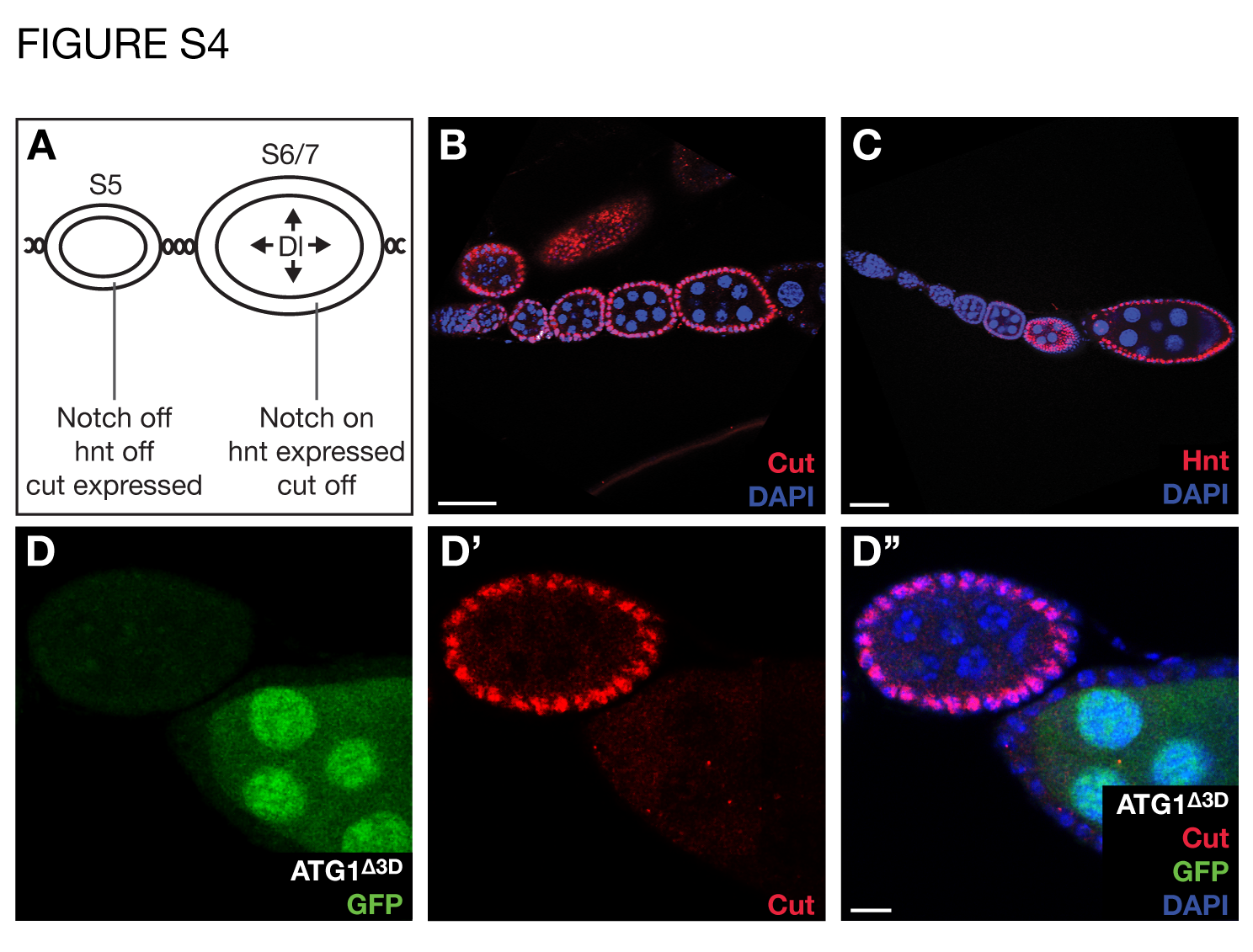

Supplement: Additional file 4 Figure S4 — Expression pattern of the Notch signaling targets Cut and Hnt. (A) Schematic representation of Notch signaling activity. Until stage 5, Delta (Dl) is not expressed by the germline, Notch is not activated in FCs, and Cut is expressed whereas Hnt is absent. By stage 6, Dl is expressed by the germline and activates Notch in FCs, Cut is downregulated, and Hnt is expressed (B) Expression of Cut starting in the germarium and continuing until stage 6. (C) Expression of Hnt is absent in early stages but expression is activated by stage 6. (D) Eggs lacking ATG1 function in both GCs and FCs (marked by the lack of GFP) show normal activation of the Notch pathway with Cut expression levels comparable to WT eggs. Anterior is to the left, posterior to the right. Scale bar: 50 μm (B-C), 20 μm (D). Genotypes: B, C: y w. D: hs flp/+; ATG1∆ 3DFRT80B/FRT80B-UbiGFP. [file 1471-213X-12-35-S4.tiff]
